# Pre-Stroke Cardiovascular Pharmacotherapy and Early Systemic Hemodynamics After Acute Ischemic Stroke: A Prospective Multicenter Cohort Study

**DOI:** 10.3390/jcm15218386

**Published:** 2026-10-28

**Authors:** Piotr Gutknecht, Bartosz G. Trzeciak, Marek Tałałaj, Krzysztof Dziewiatowski, Grzegorz Wochyń, Walenty M. Nyka, Alicja Bortkiewicz, Dariusz Świetlik, Janusz Siebert

**Affiliations:** 1 Department of Family Medicine, Medical University of Gdańsk, Dębinki 7, 80-211 Gdańsk, Poland; bartosz.trzeciak@gumed.edu.pl (B.G.T.);; 2 Department of Orthopedics, Pediatric Orthopedics and Traumatology, Centre of Postgraduate Medical Education, 05-400 Otwock, Poland; 3 Department of Neurology, 7th Navy Hospital, 80-305 Gdańsk, Poland; 4 Department of Neurology, Medical University of Gdańsk, 80-214 Gdańsk, Poland; 5 Department of Work Physiology and Ergonomics, Nofer Institute of Occupational Medicine, 91-348 Łódź, Poland; 6 Division of Biostatistics and Neural Networks, Medical University of Gdańsk, 80-211 Gdańsk, Poland

**Keywords:** acute ischemic stroke, cardiovascular pharmacotherapy, impedance cardiography, systemic hemodynamics, stroke-heart syndrome

## Abstract

**Background/Objectives:** Whether pre-stroke cardiovascular treatment is reflected in the early systemic hemodynamic phenotype after acute ischemic stroke (AIS) is uncertain. We examined associations between major medication classes, early impedance-cardiography measures, and 6-month mortality. **Methods:** This prospective multicenter cohort included 233 patients with AIS and available pre-stroke medication data. Hemodynamics were assessed within 24 h of hospital admission. Four drug–physiology pairs were prespecified: beta-blockers–heart rate, renin–angiotensin system inhibitors–systemic vascular resistance index (SVRI), calcium-channel blockers–SVRI, and loop/thiazide(-like) diuretics–thoracic fluid content (TFC). Because technical equivalence of absolute TFC output between acquisition platforms was not established, inferential TFC analyses were restricted to CardioScreen/Niccomo observations. Primary hemodynamic models used HC3 robust standard errors with Holm correction; mortality models used FDR correction. **Results:** Mean age was 73.7 ± 12.1 years, 53.2% were women, and median NIHSS was 7 [4–12]. None of the four primary associations was supported after Holm correction. Adjusted differences were +0.23 bpm for beta-blockers–heart rate, +192 dyn·s·cm^−5^·m^2^ for RAAS inhibitors–SVRI, +254 dyn·s·cm^−5^·m^2^ for calcium-channel blockers–SVRI, and −3.72 1/kΩ for diuretics–TFC in the CardioScreen/Niccomo-restricted analysis (nominal *p* = 0.013; Holm *p* = 0.053). No secondary association survived FDR correction (all *q* ≥ 0.406). Forty-seven of 208 patients (22.6%) died within 6 months; no medication class was independently associated with mortality (all *q* ≥ 0.711). **Conclusions:** Documented pre-stroke cardiovascular medication use was not robustly associated with the early systemic hemodynamic phenotype or adjusted 6-month mortality after AIS. Modest class-specific associations cannot be excluded.

## 1. Introduction

Acute ischemic stroke (AIS) is not confined to the cerebral circulation but can induce systemic cardiovascular disturbances during the earliest phase of the disease. Disruption of central autonomic networks, sympathetic activation, inflammatory signaling, and neurocardiogenic mechanisms may alter heart rate, blood pressure regulation, myocardial function, vascular tone, and fluid distribution. These interconnected manifestations are increasingly conceptualized within the stroke–heart syndrome and the broader heart–brain axis [1,2]. Non-invasive impedance cardiography (ICG) provides an opportunity to characterize several components of this systemic response simultaneously. In addition to heart rate and blood pressure, ICG-derived measures include cardiac index, stroke index, systemic vascular resistance index (SVRI), and thoracic fluid content (TFC), an impedance-derived measure related to thoracic fluid status; previous work has demonstrated the feasibility of multidimensional systemic hemodynamic assessment during acute stroke [3].

Patients enter this acute physiological state with pre-existing cardiovascular disease and documented pre-stroke pharmacotherapy. Major cardiovascular drug classes have distinct expected physiological effects: systemic beta-blockers may reduce chronotropic drive; renin–angiotensin system inhibitors and calcium-channel blockers may modify vascular resistance; and loop or thiazide (-like) diuretics may influence loading conditions and thoracic fluid content. These relationships provide biologically plausible drug–physiology pairs that can be examined using early hemodynamic measurements. Statins and antithrombotic therapies may also influence subsequent clinical outcome through vascular, thrombotic, or pleiotropic pathways that are not necessarily reflected in a single early hemodynamic variable.

Whether pre-stroke treatment-related physiological effects remain detectable after the onset of AIS is uncertain. Acute cerebral ischemia introduces a rapidly evolving environment in which autonomic activation, inflammation, neurological severity, hydration status, and acute therapeutic interventions may modify systemic cardiovascular measurements. Consequently, an early ICG assessment should not be interpreted as a direct pharmacodynamic measurement of a drug taken before stroke. Rather, it represents the integrated systemic cardiovascular state resulting from pre-existing disease and treatment superimposed on the acute response to cerebral ischemia. Determining whether documented pre-stroke medication history is nevertheless associated with a detectable class-specific hemodynamic pattern is therefore physiologically and clinically relevant.

Previous observational studies have mainly evaluated associations between pre-stroke cardiovascular treatment and stroke severity or clinical outcome, with inconsistent findings across medication classes. Pre-stroke beta-blocker use was not independently associated with ischemic stroke severity or functional outcome in a European Journal of Neurology analysis [4], whereas earlier community-based data suggested that angiotensin-converting enzyme inhibitor use might be associated with less severe stroke [5]. A systematic review of pre-stroke statin treatment reported associations with milder neurological deficits, better functional outcome, or lower mortality while emphasizing the observational nature and potential susceptibility to bias of the available evidence [6]. Among patients with atrial fibrillation, therapeutic anticoagulation before stroke has been associated with lower stroke severity and improved in-hospital outcomes [7]. Collectively, these studies suggest that treatment history may be relevant to the clinical phenotype of AIS, but they do not establish whether major cardiovascular medication classes are reflected in the early systemic hemodynamic state.

Evidence addressing intermediate physiological phenotypes is substantially more limited. A recent cohort comparing pre-stroke antihypertensive treatment groups found no material differences in dynamic cerebral autoregulation, despite selected associations with stroke severity and 3-month outcome [8]. Other work has linked premorbid beta-blocker exposure to aspects of the peripheral immune response after AIS, illustrating that pre-stroke cardiovascular therapy may interact with post-stroke physiology through pathways that are not fully captured by conventional clinical endpoints [9]. To our knowledge, major pre-stroke cardiovascular medication classes have not been systematically examined in relation to a multidimensional assessment of systemic hemodynamics by ICG during the first 24 h of hospitalization for AIS.

We therefore evaluated whether documented pre-stroke use of major cardiovascular medication classes was associated with prespecified early systemic hemodynamic measures and, secondarily, with 6-month all-cause mortality. Four primary drug–physiology relationships were defined on physiological grounds: systemic beta-blocker use with heart rate, renin–angiotensin system inhibitor use with SVRI, calcium-channel blocker use with SVRI, and loop/thiazide(-like) diuretic use with TFC. Our primary objective was not to quantify the direct pharmacodynamic effect of individual drugs, but to determine whether documented pre-stroke treatment was associated with a detectable class-specific physiological signature within the complex acute systemic response to cerebral ischemia.

## 2. Materials and Methods

### 2.1. Study Design and Population

This prospective multicenter observational study used a prospectively maintained database of consecutive patients with acute stroke recruited at three neurological departments in Poland and undergoing standardized clinical and non-invasive hemodynamic assessment. Stroke diagnosis was established by neurological assessment supported by computed tomography and/or magnetic resonance imaging. The source cohort comprised 277 patients; 10 patients with hemorrhagic stroke were excluded, leaving 267 patients with confirmed acute ischemic stroke (AIS). The present pharmacotherapy analysis included 233 patients with available pre-stroke medication information; 34 patients with unavailable medication status were excluded from exposure analyses. Age at stroke was calculated from date of birth and stroke date. Baseline neurological severity was assessed using the National Institutes of Health Stroke Scale (NIHSS). Cardiovascular comorbidities were identified from medical history and available clinical documentation and included hypertension, diabetes mellitus, ischemic heart disease, atrial fibrillation, heart failure, kidney disease, dyslipidemia, and prior stroke. No formal sample-size calculation was performed specifically for this pharmacotherapy analysis; all eligible patients with AIS and available pre-stroke medication data were included. Reporting follows the Strengthening the Reporting of Observational Studies in Epidemiology (STROBE) statement for cohort studies [10]. The original study approval was granted by the Independent Bioethics Commission for Scientific Research at the Medical University of Gdańsk (No. NKBBN/64/2014; 4 March 2014) and was maintained in the amendment No. NKBBN/64-451/2015 dated 22 October 2015.

### 2.2. Pre-Stroke Pharmacotherapy Ascertainment and Exposure Definitions

Pre-stroke medication use was derived from medication histories recorded in the study database, incorporating hospital documentation and available pre-admission information. The structured medication list was used as the primary source, while unequivocal free-text drug names were used to supplement class assignment when drug identity was clear. Existing binary medication-class indicators were used for consistency checks only; an uncorroborated binary positive did not define exposure in the primary analysis. Ophthalmic beta-blockers were excluded from systemic beta-blocker exposure.

The four prespecified primary exposure classes were systemic beta-blockers, renin–angiotensin–aldosterone system (RAAS) inhibitors, defined as angiotensin-converting enzyme inhibitors or angiotensin receptor blockers, calcium-channel blockers (CCBs), and loop/thiazide or thiazide-like diuretics. Spironolactone was excluded from the primary diuretic definition. Statins and acetylsalicylic acid (ASA) were evaluated as secondary prognostic exposures. Among patients with atrial fibrillation, pre-stroke anticoagulation was assessed using the dedicated source variable, with medication-list coding used as a sensitivity definition. Dose, treatment duration, adherence, timing of the last pre-stroke dose, and drug concentrations were not available. Accordingly, medication exposure represents documented pre-stroke treatment history rather than verified active pharmacological exposure at the time of hemodynamic assessment.

### 2.3. Hemodynamic Assessment

Systemic hemodynamics were assessed non-invasively by thoracic impedance cardiography under a standardized bedside protocol. The examination used in the present analysis was performed within 24 h of hospital admission with patients resting in the supine position and surface electrodes positioned according to the applicable acquisition protocol. Each ICG recording lasted for at least 10 min; the first 2 min after initiation of the examination were treated as a stabilization period, and hemodynamic values were considered thereafter. Exact onset-to-ICG and admission-to-ICG intervals were not available. CardioScreen/Niccomo™ (Medis Medizinische Messtechnik GmbH, Ilmenau, Germany) was used at two participating centers and PhysioFlow ICG (Manatec Biomedical, Folschviller, France) at one center; all measurements for an individual participant were obtained using the same system. Core variables were heart rate (HR), mean arterial pressure (MAP), cardiac index (CI), stroke index (SI), systemic vascular resistance index (SVRI), and thoracic fluid content (TFC). HR, MAP, CI, SI, and SVRI were available for all 233 patients. Because technical equivalence of absolute TFC output between the two systems had not been established, inferential TFC analyses were restricted to CardioScreen/Niccomo observations.

### 2.4. Six-Month Mortality Ascertainment

The secondary clinical endpoint was all-cause mortality during the index hospitalization or within 6 months after AIS. Vital status was determined from hospital and institutional records and structured follow-up with patients or relatives, supplemented by available administrative information. Patients known to be alive beyond 6 months were classified as survivors. Because dates of death were incomplete for a small number of deaths, mortality was analyzed as a binary 6-month endpoint rather than as the primary time-to-event outcome.

### 2.5. Statistical Analysis

The statistical analysis plan was finalized before the prespecified inferential analyses were performed. The four primary drug–physiology hypotheses, principal covariate sets, and multiplicity procedures were specified before examination of the primary inferential results. Continuous variables are summarized as mean ± standard deviation or median [interquartile range], and categorical variables as counts and percentages. Four primary drug–physiology hypotheses were defined a priori: systemic beta-blocker use with HR, RAAS inhibitor use with SVRI, CCB use with SVRI, and loop/thiazide (-like) diuretic use with TFC. Primary associations were estimated using multivariable linear regression with HC3 heteroskedasticity-robust standard errors and are reported as adjusted mean differences with 95% confidence intervals (CIs). All tests were two-sided, and family-wise error across the four primary hypotheses was controlled using the Holm procedure.

All primary hemodynamic models included age, sex, and adjustment for the three participating centers together with prespecified indication-related covariates: hypertension, atrial fibrillation, ischemic heart disease, and heart failure for beta-blockers; hypertension, heart failure, ischemic heart disease, diabetes, and kidney disease for RAAS inhibitors; hypertension, ischemic heart disease, and atrial fibrillation for CCBs; and hypertension, heart failure, and kidney disease for diuretics. The two ICG systems were center-specific. Non-TFC models used observations from both systems, whereas all inferential TFC models were restricted to CardioScreen/Niccomo observations. Admission NIHSS was added in severity-adjusted sensitivity models. Secondary hemodynamic analyses evaluated HR, MAP, CI, SI, SVRI, and TFC across the four primary medication classes, excluding the four primary cells; Benjamini–Hochberg false-discovery-rate (FDR) correction was applied across these 20 comparisons. Analytic sample size for each regression model refers to the complete comparison sample containing both medication-exposed and medication-unexposed participants with the required outcome and covariate data. Center was included as a categorical adjustment variable (UCK Gdańsk, 7th Navy Hospital Gdańsk, and IPiN Warsaw).

Six-month mortality was analyzed using separate multivariable logistic regression models for systemic beta-blockers, RAAS inhibitors, CCBs, loop/thiazide(-like) diuretics, statins, and ASA. Principal prognostic models included age, sex, admission NIHSS, and prespecified exposure-specific confounders: atrial fibrillation and heart failure for beta-blockers; hypertension and heart failure for RAAS inhibitors; hypertension and ischemic heart disease for CCBs; hypertension and heart failure for diuretics; ischemic heart disease and diabetes for statins; and ischemic heart disease and prior stroke for ASA. Results are reported as odds ratios (ORs) with 95% CIs, with BH-FDR correction across the six full-cohort exposure tests. Pre-stroke anticoagulation was examined separately in the prespecified atrial-fibrillation subgroup and was considered exploratory.

Medication exposures were not imputed. Hemodynamic analyses used variable-specific available cases, and mortality analyses included participants with known 6-month vital status. Complete-case analysis was used for model covariates, with model-specific analytic sample sizes reported in the corresponding tables; no covariate imputation was performed. Selection associated with unavailable medication history and missing follow-up was evaluated using standardized mean differences (Tables S1 and S2). Secondary hemodynamic findings and their sensitivity analyses are presented in Tables S3 and S4, respectively. A stabilized inverse-probability-of-observation weighting sensitivity analysis with a ridge-penalized follow-up observation model was performed to address incomplete follow-up and separation; methodological details are provided in Table S5. Additional sensitivity analyses assessed influential observations and nonlinear age effects where appropriate. No stepwise variable selection or exclusion based solely on extreme hemodynamic values was used. Analyses were performed in Python 3.13.5 (Python Software Foundation, Beaverton, OR, USA) using statsmodels 0.14.6 (https://www.statsmodels.org/, accessed on 21 October 2026) and SciPy 1.17.0 (https://scipy.org/, accessed on 21 October 2026). Statistical support was defined as Holm-adjusted *p* < 0.05 for the primary family and *q* < 0.05 for FDR-controlled secondary families; interpretation emphasized effect estimates and 95% CIs. Age and admission NIHSS were modeled continuously. A center-stratified bootstrap that refitted observation weights in each replicate yielded confidence intervals encompassing the null for all six exposures (Table S5).

OpenAI ChatGPT (GPT-5.6 Sol; OpenAI, San Francisco, CA, USA) was used to assist with language editing, manuscript organization, and preparation and checking of statistical code. No study data were generated or altered by the AI tool, and no statistical result or clinical interpretation was accepted without author verification. All analyses were performed on the authors’ data, and the authors independently reviewed and verified the statistical code, numerical outputs, references, interpretation, and final manuscript content. The authors take full responsibility for the content of the manuscript.

## 3. Results

### 3.1. Study Cohort and Pre-Stroke Pharmacotherapy

**Figure 1:**
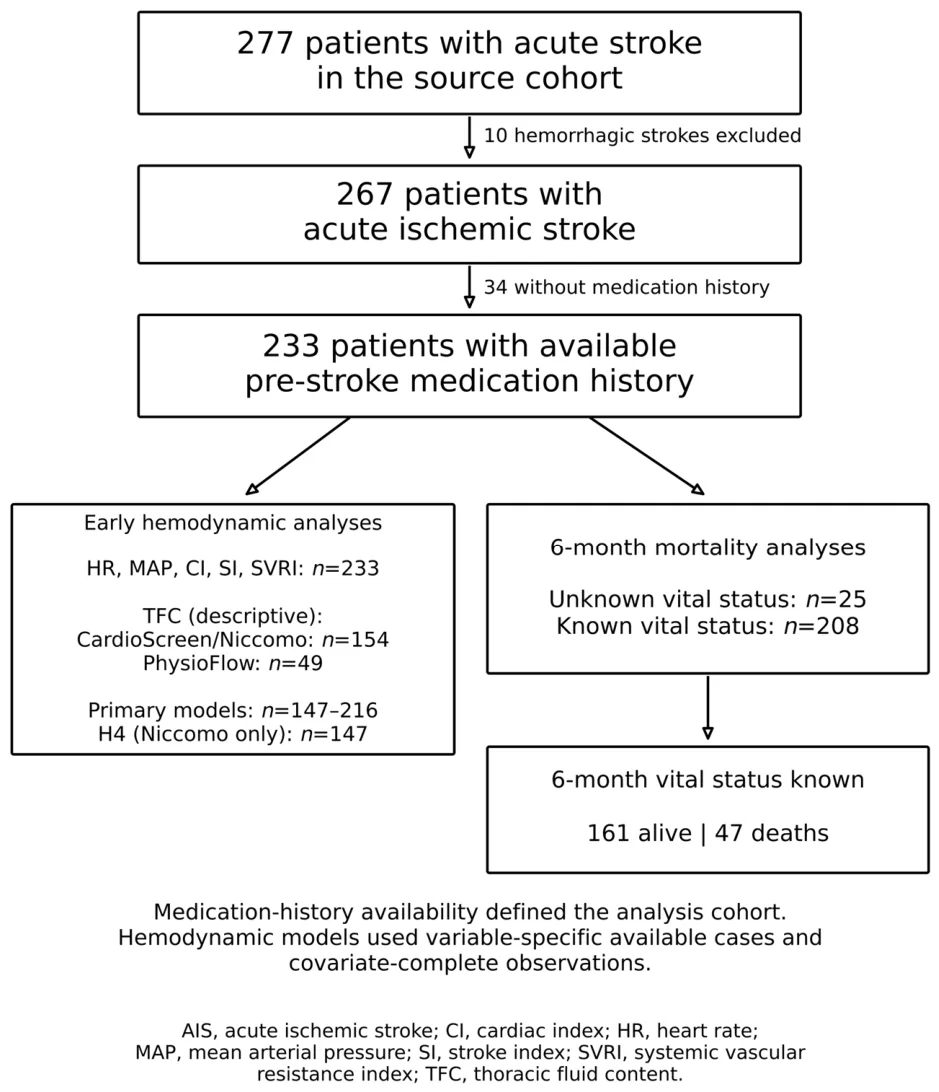
Study flow and analytic cohorts. The source cohort comprised 277 patients with acute stroke. After exclusion of 10 hemorrhagic strokes, 267 patients with AIS remained; 233 had available pre-stroke medication information and formed the medication-analysis cohort. Hemodynamic models used variable-specific available cases and prespecified covariate-complete observations. Primary adjusted model sizes ranged from *n* = 147 to *n* = 216; the H4 diuretic–TFC model was restricted to CardioScreen/Niccomo observations (*n* = 147). Six-month vital status was known for 208 patients (161 survivors and 47 deaths). AIS, acute ischemic stroke; CI, cardiac index; HR, heart rate; MAP, mean arterial pressure; SI, stroke index; SVRI, systemic vascular resistance index; TFC, thoracic fluid content.

Of 277 patients in the source acute-stroke cohort, 10 with hemorrhagic stroke were excluded, leaving 267 patients with AIS. Pre-stroke medication information was available for 233 patients, who constituted the medication-analysis cohort (Figure 1). Their mean age was 73.7 ± 12.1 years, 124 (53.2%) were women, and the median admission NIHSS was 7 [4–12]. Hypertension was present in 179/232 patients (77.2%), ischemic heart disease in 72/221 (32.6%), atrial fibrillation in 74/224 (33.0%), diabetes mellitus in 54/227 (23.8%), heart failure in 33/226 (14.6%), and kidney disease in 17/227 (7.5%) (Table 1). Mean early HR was 75.2 ± 17.9 bpm, MAP 97.2 ± 13.9 mmHg, CI 2.98 ± 0.95 L/min/m^2^, SI 41.1 ± 12.7 mL/m^2^, and SVRI 2902 ± 1322 dyn·s·cm^−5^·m^2^. Because absolute TFC values were not pooled across acquisition platforms, TFC is reported descriptively by device: mean TFC was 33.9 ± 7.9 1/kΩ in 154 CardioScreen/Niccomo patients and 20.9 ± 12.1 1/kΩ in 49 PhysioFlow patients.

RAAS inhibitors were used by 111/233 patients (47.6%), systemic beta-blockers by 98 (42.1%), ASA by 96 (41.2%), statins by 87 (37.3%), loop/thiazide(-like) diuretics by 71 (30.5%), and CCBs by 50 (21.5%). Among 74 patients with atrial fibrillation, 35 (47.3%) were classified as receiving pre-stroke anticoagulation using the dedicated source variable. Medication-data availability and 6-month follow-up completeness are summarized in Tables S1 and S2.

### 3.2. Primary Hemodynamic Associations

None of the four prespecified drug–physiology associations was supported after Holm correction (Table 2). For systemic beta-blocker use, the adjusted difference in HR was +0.23 bpm (95% CI −5.13 to +5.58; raw *p* = 0.934; Holm-adjusted *p* = 1.000; *n* = 214). RAAS inhibitor use was associated with an adjusted SVRI difference of +192 dyn·s·cm^−5^·m^2^ (95% CI −209 to +592; raw *p* = 0.346; Holm *p* = 1.000; *n* = 216). For CCB use, the adjusted SVRI difference was +254 dyn·s·cm^−5^·m^2^ (95% CI −279 to +787; raw *p* = 0.348; Holm *p* = 1.000; *n* = 216). In the CardioScreen/Niccomo-restricted H4 analysis, loop/thiazide(-like) diuretic use was associated with lower TFC, with an adjusted difference of −3.72 1/kΩ (95% CI −6.64 to −0.79; nominal *p* = 0.013; Holm-adjusted *p* = 0.053; *n* = 147). The H4 analysis comprised 43 diuretic-exposed and 104 unexposed participants. Although the estimate was nominally significant before multiplicity correction, it did not meet the prespecified criterion for statistical support within the four-hypothesis primary family.

Additional adjustment for admission NIHSS attenuated the H4 estimate to −3.22 1/kΩ (95% CI −6.17 to −0.27; *p* = 0.033; *n* = 147). Further sensitivities adjusting for other primary medication classes or excluding influential observations showed variation in the effect estimate, without establishing multiplicity-controlled evidence for H4 (Table S4). Overall, none of the four primary hypotheses was supported after correction for multiple testing.

Adjusted mean differences were estimated using prespecified drug-specific multivariable linear regression models with HC3 robust standard errors. The Holm procedure controlled the family-wise error across the four primary hypotheses. Model *N* represents the total complete-case analytic sample, including both medication-exposed and medication-unexposed participants. For H4, *n* = 147 comprised 43 diuretic-exposed and 104 unexposed participants. TFC analyses were restricted to CardioScreen/Niccomo observations because technical equivalence of absolute TFC output between acquisition systems was not established. HR, heart rate; RAAS, renin–angiotensin–aldosterone system; SVRI, systemic vascular resistance index; TFC, thoracic fluid content.

### 3.3. Secondary Hemodynamic Associations

Across the 20 secondary drug–hemodynamic comparisons, none remained supported after Benjamini–Hochberg FDR correction (all *q* ≥ 0.406; Table S3). Two associations had nominal *p* < 0.05: RAAS inhibitor use was associated with lower HR (adjusted difference −5.63 bpm, 95% CI −11.02 to −0.24; *p* = 0.041; *q* = 0.406), and loop/thiazide(-like) diuretic use was associated with lower MAP (adjusted difference −4.36 mmHg, 95% CI −8.38 to −0.35; *p* = 0.033; *q* = 0.406). Neither association met the prespecified FDR-controlled criterion for statistical support. All secondary TFC comparisons were restricted to CardioScreen/Niccomo observations, and none was nominally significant after this restriction. Severity-adjusted and influence sensitivity analyses did not alter the overall inference that no secondary association was FDR-supported.

### 3.4. Six-Month Mortality

Six-month vital status was known for 208/233 patients (89.3%); 47 patients (22.6%) died within 6 months, including 22 in-hospital deaths, and 161 were known to be alive beyond 6 months. In the principal NIHSS-adjusted logistic regression models, none of the six full-cohort medication classes was independently associated with 6-month all-cause mortality after FDR correction (Table 3). Adjusted ORs were 1.22 for systemic beta-blocker use (95% CI 0.52–2.84; *p* = 0.646; *q* = 0.806), 0.76 for RAAS inhibitor use (0.31–1.86; *p* = 0.546; *q* = 0.806), 0.41 for CCB use (0.13–1.26; *p* = 0.119; *q* = 0.711), 0.82 for loop/thiazide(-like) diuretic use (0.32–2.10; *p* = 0.672; *q* = 0.806), 0.80 for statin use (0.32–2.00; *p* = 0.636; *q* = 0.806), and 1.02 for ASA use (0.40–2.58; *p* = 0.968; *q* = 0.968). A nominal association between CCB use and lower mortality in the model without admission NIHSS (OR 0.36, 95% CI 0.14–0.95; *p* = 0.038) attenuated after adjustment for neurological severity and was not FDR-supported. Inverse-probability-of-observation weighting for incomplete follow-up produced materially similar estimates (Table S5).

In the prespecified atrial-fibrillation subgroup with known 6-month outcome (*n* = 67; 26 deaths), mortality occurred in 10/34 patients (29.4%) classified as anticoagulated before stroke and 16/33 (48.5%) classified as not anticoagulated. After adjustment for age, sex, and admission NIHSS, the OR was 0.64 (95% CI 0.19–2.14; *p* = 0.473). This exploratory estimate was imprecise and compatible with no association. Sensitivity analyses allowing for nonlinear age effects likewise did not materially alter the medication-effect estimates (Tables S5 and S6).

Principal models included age, sex, admission NIHSS, and prespecified exposure-specific indication/confounding variables. Model *N* represents the complete-case sample for each exposure-specific model; variation in *N* reflects missing covariate data. BH-FDR correction was applied across the six full-cohort exposure tests. ASA, acetylsalicylic acid; BH-FDR, Benjamini–Hochberg false-discovery rate; NIHSS, National Institutes of Health Stroke Scale; OR, odds ratio; RAAS, renin–angiotensin–aldosterone system.

## 4. Discussion

This prospective multicenter study examined whether documented cardiovascular medications used before AIS were reflected in systemic hemodynamics during the first 24 h of hospitalization and, secondarily, in 6-month mortality. None of the four prespecified drug–physiology associations met the multiplicity-adjusted criterion for statistical support, although the H4 diuretic–TFC estimate approached this threshold (Holm *p* = 0.053). Similarly, none of the secondary drug–hemodynamic associations remained supported after FDR correction, and no medication class was independently associated with 6-month all-cause mortality in the principal NIHSS-adjusted models. These findings do not demonstrate pharmacological equivalence between treated and untreated patients, nor do they imply absence of expected drug effects. Rather, within the precision and exposure characterization available in this cohort, pre-stroke treatment history alone did not identify a robust class-specific systemic hemodynamic phenotype during the acute phase of cerebral ischemia.

The absence of a strong medication-specific signal should be interpreted in the context of the marked physiological perturbation caused by acute stroke. Cerebral ischemia may disrupt central autonomic networks and trigger sympathetic activation, altered parasympathetic control, inflammatory signaling, arrhythmia, myocardial dysfunction, and changes in vascular regulation, phenomena encompassed within the stroke–heart syndrome and post-stroke autonomic dysfunction [1,2,11,12,13,14]. Sympathetic overactivation and catecholamine signaling are also proposed contributors to neurogenic cardiac injury, and post-stroke cardiovascular complications have prognostic relevance [15,16]. Consequently, the cardiovascular state measured during the first day of hospitalization represents the combined effect of pre-existing cardiovascular disease and treatment, the acute neurological insult, and early clinical management. Acute medications, hydration, evolving neurological injury, and changes in autonomic tone may all modify heart rate, vascular resistance, cardiac output, and thoracic fluid distribution. Thus, early ICG should not be interpreted as a direct pharmacodynamic assay of a drug taken before stroke. The relevant question is whether pre-stroke treatment history leaves a detectable physiological signature despite this competing acute biological variability.

This framework is particularly relevant to the beta-blocker–HR hypothesis. A lower heart rate among beta-blocker users might be expected under stable conditions, yet the adjusted difference in HR was essentially null. This result should not be interpreted as evidence that beta-blockade had no biological effect. Dose, treatment duration, adherence, timing of the last dose, and circulating drug concentrations were unavailable, so recorded use represented treatment history rather than verified active exposure at the time of ICG. Moreover, beta-blockers were prescribed for conditions such as atrial fibrillation, ischemic heart disease, and heart failure, all of which may independently influence heart rate and cardiac performance. Previous observational studies have likewise found no consistent association between pre-stroke beta-blocker use and stroke severity, disability, or mortality [4,17]. Premorbid beta-blocker exposure has also been associated with differences in the peripheral immune response without clear functional benefit, suggesting that treatment history may interact with post-stroke biology through pathways not captured by a single hemodynamic endpoint [9].

The RAAS inhibitor–SVRI and CCB–SVRI hypotheses were also not supported. Although these drug classes can influence vascular tone under stable conditions, systemic vascular resistance during acute AIS is likely determined by multiple interacting processes, including baseline hypertension, autonomic activation, cardiac output, volume status, and acute treatment. Observational studies of pre-stroke renin–angiotensin system blockade have reported heterogeneous associations with stroke severity and outcome [5,18,19,20]. A post hoc analysis of BP TARGET reported lower baseline NIHSS among patients receiving its study-defined RAS-inhibitor antihypertensive category but no association with favorable functional outcome [19], while other observational data have suggested favorable associations with ACE-inhibitor-based pre-stroke regimens [20]. Lakatos et al. found no material differences in dynamic cerebral autoregulation across antihypertensive treatment groups despite selected associations with stroke severity and 3-month outcome [8]. Our findings extend this uncertainty to systemic hemodynamic phenotypes measured by ICG.

The diuretic–TFC hypothesis showed the strongest numerical signal. In the CardioScreen/Niccomo-restricted primary analysis, pre-stroke loop/thiazide(-like) diuretic use was associated with lower TFC (adjusted difference −3.72 1/kΩ; nominal *p* = 0.013), but the association did not meet the Holm-corrected threshold (*p* = 0.053). Additional adjustment for admission NIHSS modestly attenuated the estimate (−3.22 1/kΩ; *p* = 0.033). This pattern is compatible with a possible modest class-specific difference, but it is not sufficient to establish a reproducible treatment-related effect. TFC reflects thoracic electrical conductivity and is influenced by fluid distribution and loading conditions; therefore, any pre-stroke volume-related effect of diuretic therapy may be modified substantially by the acute clinical state. The appropriate interpretation is uncertainty rather than either confirmation or exclusion of a diuretic-associated physiological signal.

The secondary hemodynamic analyses support the same cautious interpretation. RAAS inhibitor use was nominally associated with lower HR and diuretic use with lower MAP, but neither finding survived FDR correction. Following restriction of TFC analyses to the technically comparable CardioScreen/Niccomo dataset, the previously observed nominal association between CCB use and lower TFC was no longer present. Thus, the exploratory findings do not provide evidence for a consistent cross-variable pattern attributable to a specific medication class. Taken together, the results suggest that the acute systemic hemodynamic phenotype after AIS is likely shaped by multiple overlapping determinants rather than by pre-stroke pharmacotherapy alone.

The mortality analyses similarly showed no independent association between the six evaluated medication classes and 6-month all-cause mortality after adjustment for neurological severity and relevant clinical covariates. The nominal association between CCB use and lower mortality in the model without NIHSS attenuated after adjustment for stroke severity. Previous studies have reported associations between pre-stroke statin or antiplatelet therapy and selected measures of severity or outcome, but the observational literature remains heterogeneous [6,21,22,23,24]. More comprehensive pre-stroke control of major vascular risk factors has also been associated with better functional outcome [25]. Among patients with atrial fibrillation, therapeutic anticoagulation before stroke has been associated with lower stroke severity and in-hospital mortality [7]. Our AF subgroup showed numerically lower mortality among anticoagulated patients, but the adjusted estimate was imprecise and compatible with no association. Sensitivity analyses for incomplete follow-up were consistent with the principal mortality findings.

From a clinical perspective, these findings suggest that pre-stroke medication history alone provides limited information about the early systemic hemodynamic profile after AIS. The width of several confidence intervals means that modest class-specific effects cannot be excluded, particularly for less common exposures.

Several strengths of the study should be noted. The four primary drug–physiology relationships were prespecified on physiological grounds, the analysis emphasized effect estimates and confidence intervals while controlling multiplicity, and multivariable models incorporated indication-related covariates. The use of multidimensional ICG measurements also allowed evaluation of cardiac, vascular, and volume-related components of systemic cardiovascular function rather than reliance on brachial blood pressure alone.

The study also has important limitations. Residual confounding by indication, comorbidity, co-medication, stroke mechanism, and acute treatment is unavoidable in an observational cohort. Dose, treatment duration, adherence, timing of the last pre-stroke dose, and drug concentrations were unavailable, so documented medication use should not be interpreted as verified active pharmacological exposure at the time of ICG. Although examinations were standardized and performed within 24 h of hospital admission, exact onset-to-ICG and admission-to-ICG intervals were unavailable. Medication history was unavailable in 34 patients, and 25 patients lacked known 6-month outcomes, so selection bias cannot be excluded. The acquisition platform was center-specific, so platform and center effects on absolute impedance-derived values could not be separated; inferential TFC analyses were therefore restricted to CardioScreen/Niccomo observations. Finally, several exposure groups were modest in size, and the study was not powered specifically for class-specific pharmacotherapy effects. Missing medication history and follow-up were unevenly distributed by center, which may have contributed to selection bias despite the sensitivity analyses.

Future prospective studies should incorporate more detailed medication exposure data, precise measurement timing, and repeated hemodynamic assessment. Larger cohorts with harmonized acquisition protocols may help determine whether medication-related physiological patterns can be identified more reliably.

## 5. Conclusions

In this prospective multicenter cohort, documented pre-stroke use of major cardiovascular medication classes was not robustly associated with prespecified systemic hemodynamic measures obtained during the first 24 h of hospitalization for acute ischemic stroke or with adjusted 6-month all-cause mortality. The diuretic–TFC association was nominally significant in the primary and NIHSS-adjusted analyses but did not meet the prespecified Holm-corrected threshold in the primary analysis.

These findings should not be interpreted as evidence that the evaluated drug classes lack their expected cardiovascular effects. Rather, pre-stroke medication history alone appears to provide limited information about the integrated systemic hemodynamic state during the acute phase of stroke. Larger prospective studies with detailed pharmacological exposure data, precise measurement timing, harmonized acquisition protocols, and repeated systemic and cerebral physiological assessments are needed to determine whether medication-related hemodynamic signatures can be identified more reliably.

## Figures and Tables

**Table 1:** Clinical characteristics, early systemic hemodynamics, and pre-stroke pharmacotherapy in the medication-analysis cohort.

Variable	Summary
Age, years	73.7 ± 12.1
Female sex	124/233 (53.2%)
NIHSS on admission	7 [4–12]
Hypertension	179/232 (77.2%)
Diabetes mellitus	54/227 (23.8%)
Ischemic heart disease	72/221 (32.6%)
Atrial fibrillation	74/224 (33.0%)
Heart failure	33/226 (14.6%)
Kidney disease	17/227 (7.5%)
Prior stroke	53/221 (24.0%)
Heart rate, bpm	75.2 ± 17.9
Mean arterial pressure, mmHg	97.2 ± 13.9
Cardiac index, L/min/m^2^	2.98 ± 0.95
Stroke index, mL/m^2^	41.1 ± 12.7
Systemic vascular resistance index, dyn·s·cm^−5^·m^2^	2902 ± 1322
Thoracic fluid content, CardioScreen/Niccomo, 1/kΩ	33.9 ± 7.9 (*n* = 154)
Thoracic fluid content, PhysioFlow, 1/kΩ	20.9 ± 12.1 (*n* = 49)
Systemic beta-blocker	98/233 (42.1%)
RAAS inhibitor	111/233 (47.6%)
Calcium-channel blocker	50/233 (21.5%)
Loop/thiazide(-like) diuretic	71/233 (30.5%)
Statin	87/233 (37.3%)
ASA	96/233 (41.2%)
Known 6-month vital status	208/233 (89.3%)
6-month all-cause mortality	47/208 (22.6%)

Note: Values are mean ± SD, median [IQR], or *n*/*N* (%), as appropriate. Denominators reflect available data. TFC values are reported separately by acquisition platform because absolute outputs were not pooled across devices for inferential analyses. ASA, acetylsalicylic acid; NIHSS, National Institutes of Health Stroke Scale; RAAS, renin–angiotensin–aldosterone system.

**Table 2:** Prespecified adjusted associations between pre-stroke cardiovascular pharmacotherapy and early systemic hemodynamics.

Exposure → Outcome	*N*	Adjusted Difference	95% CI	Raw *p*	Holm-Adjusted *p*
Systemic beta-blocker → HR, bpm	214	+0.23	−5.13 to +5.58	0.934	1.000
RAAS inhibitor → SVRI, dyn·s·cm^−5^·m^2^	216	+191.73	−208.64 to +592.10	0.346	1.000
Calcium-channel blocker → SVRI, dyn·s·cm^−5^·m^2^	216	+253.96	−278.60 to +786.51	0.348	1.000
Loop/thiazide(-like) diuretic → TFC, 1/kΩ (CardioScreen/Niccomo only)	147	−3.72	−6.64 to −0.79	0.013	0.053

**Table 3:** Principal adjusted associations between pre-stroke cardiovascular pharmacotherapy and 6-month all-cause mortality.

Exposure	Model *N*	Deaths	Adjusted OR	95% CI	*p*	BH-FDR *q*
Systemic beta-blocker	197	47	1.22	0.52–2.84	0.646	0.806
RAAS inhibitor	202	47	0.76	0.31–1.86	0.546	0.806
Calcium-channel blocker	196	47	0.41	0.13–1.26	0.119	0.711
Loop/thiazide(-like) diuretic	202	47	0.82	0.32–2.10	0.672	0.806
Statin	194	47	0.80	0.32–2.00	0.636	0.806
ASA	189	44	1.02	0.40–2.58	0.968	0.968

## Data Availability

Individual-level study data are not publicly available because they contain clinical participant information and are subject to ethical and data-protection restrictions. De-identified data may be made available by the corresponding author on reasonable request, subject to applicable institutional and ethical approvals.

## Supplementary Materials

The following supporting information can be downloaded at: https://www.mdpi.com/article/10.3390/jcm15218386/s1, Table S1, Medication-data selection: included *n* = 233 vs. medication history unavailable *n* = 34; Table S2, Follow-up selection within the medication cohort: known *n* = 208 vs. unknown *n* = 25; Table S3, Secondary adjusted associations between pre-stroke medication classes and early systemic hemodynamics; Table S4, Hemodynamic sensitivity analyses; Table S5, Six-month mortality: raw contrasts and sensitivity analyses; Table S6, Exploratory pre-stroke anticoagulation analyses among patients with atrial fibrillation.
