# Supplementary material for: Pre-Stroke Cardiovascular Pharmacotherapy and Early Systemic Hemodynamics After Acute Ischemic Stroke: A Prospective Multicenter Cohort Study
Source: J Clin Med. 2026 Oct 28;15(21):8386. doi: 10.3390/jcm15218386 (PMC13764278; doi:10.3390/jcm15218386)
Supplement: Supplementary file 1 [file jcm-15-08386-s001.zip › jcm-4611757-supplementary.pdf]

Supplementary Materials

Pre-Stroke Cardiovascular Pharmacotherapy and Early Systemic Hemodynamics After Acute Ischemic Stroke: A Prospective Multicenter Cohort Study.

Tables S1–S6. All values derive from the analysis workflow used for the revised manuscript.

Supplementary Table S1. Medication-data selection: included n=233 vs medication history unavailable n=34

Variable	Included N	Included summary	Unavailable N	Unavailable summary	Signed SMD
Age, years	233	73.7 ± 12.1	34	72.4 ± 13.0	+0.106
Female sex	233	124/233 (53.2%)	34	14/34 (41.2%)	+0.243
NIHSS on admission	233	9.2 ± 6.6	34	10.1 ± 5.8	-0.147
Hypertension	232	179/232 (77.2%)	31	18/31 (58.1%)	+0.417
Diabetes mellitus	227	54/227 (23.8%)	32	8/32 (25.0%)	-0.028
Ischemic heart disease	221	72/221 (32.6%)	30	11/30 (36.7%)	-0.086
Atrial fibrillation	224	74/224 (33.0%)	30	8/30 (26.7%)	+0.140
Heart failure	226	33/226 (14.6%)	30	3/30 (10.0%)	+0.140
Kidney disease	227	17/227 (7.5%)	29	4/29 (13.8%)	-0.206
Prior stroke	221	53/221 (24.0%)	27	7/27 (25.9%)	-0.045
HR1, bpm	233	75.2 ± 17.9	34	78.6 ± 21.0	-0.175
MAP1, mmHg	233	97.2 ± 13.9	34	100.3 ± 16.3	-0.202
CI1, L/min/m²	233	2.98 ± 0.95	34	2.91 ± 0.90	+0.080
SI1, mL/m²	233	41.1 ± 12.7	34	39.0 ± 14.2	+0.155
SVRI1	233	2902 ± 1322	34	3014 ± 1209	-0.088

SMDs are descriptive. Denominators for binary variables exclude unavailable/blank values. Pooled TFC summaries and TFC SMDs are omitted from Tables S1 and S2 because absolute TFC outputs were platform-dependent.

Supplementary Table S2. Follow-up selection within the medication cohort: known n=208 vs unknown n=25

Variable	Known N	Known summary	Unknown N	Unknown summary	Signed SMD
Age, years	208	73.7 ± 12.2	25	73.8 ± 12.0	-0.013
Female sex	208	114/208 (54.8%)	25	10/25 (40.0%)	+0.300
NIHSS on admission	208	9.2 ± 6.7	25	9.4 ± 6.3	-0.029
Hypertension	207	159/207 (76.8%)	25	20/25 (80.0%)	-0.078
Diabetes mellitus	202	49/202 (24.3%)	25	5/25 (20.0%)	+0.103
Ischemic heart disease	196	63/196 (32.1%)	25	9/25 (36.0%)	-0.081
Atrial fibrillation	199	67/199 (33.7%)	25	7/25 (28.0%)	+0.123
Heart failure	202	31/202 (15.3%)	24	2/24 (8.3%)	+0.218
Kidney disease	202	16/202 (7.9%)	25	1/25 (4.0%)	+0.166
Prior stroke	196	46/196 (23.5%)	25	7/25 (28.0%)	-0.104
HR1, bpm	208	75.2 ± 17.5	25	75.2 ± 21.5	-0.003
MAP1, mmHg	208	97.1 ± 13.5	25	98.6 ± 16.9	-0.101
CI1, L/min/m²	208	2.96 ± 0.96	25	3.18 ± 0.88	-0.237
SI1, mL/m²	208	40.8 ± 12.9	25	43.4 ± 10.5	-0.221
SVRI1	208	2924 ± 1359	25	2717 ± 963	+0.176
Systemic beta-blocker	208	88/208 (42.3%)	25	10/25 (40.0%)	+0.047
RAAS inhibitor	208	98/208 (47.1%)	25	13/25 (52.0%)	-0.098
Calcium-channel blocker	208	47/208 (22.6%)	25	3/25 (12.0%)	+0.283
Loop/thiazide(-like) diuretic	208	63/208 (30.3%)	25	8/25 (32.0%)	-0.037
Statin	208	78/208 (37.5%)	25	9/25 (36.0%)	+0.031
ASA	208	86/208 (41.3%)	25	10/25 (40.0%)	+0.027
Canonical anticoagulant	208	40/208 (19.2%)	25	2/25 (8.0%)	+0.332

SMDs are descriptive; no significance testing was used for selection comparisons. Pooled TFC summaries and TFC SMDs are omitted from Tables S1 and S2 because absolute TFC outputs were platform-dependent.

Supplementary Table S3. Secondary adjusted associations between pre-stroke medication classes and early systemic hemodynamics

Exposure	Outcome	N	Exposed raw mean ± SD	Unexposed raw mean ± SD	Adjusted difference	95% CI	P	BH-FDR q
Systemic beta-blocker	MAP	214	96.74 ± 13.47	97.57 ± 14.36	−1.48 mmHg	−5.37 to +2.42	0.455	0.906
Systemic beta-blocker	CI	214	2.99 ± 1.09	2.98 ± 0.90	+0.05 L/min/m²	−0.29 to +0.39	0.778	0.906
Systemic beta-blocker	SI	214	40.40 ± 13.35	41.45 ± 12.67	+0.65 mL/m²	−3.54 to +4.83	0.761	0.906
Systemic beta-blocker	SVRI	214	3017.23 ± 1610.09	2857.50 ± 1177.13	+100.68 dyn·s·cm^{-5}·m²	−352.15 to +553.51	0.662	0.906
Systemic beta-blocker	TFC	137	32.83 ± 7.89	34.20 ± 8.13	−2.56 1/kΩ	−5.73 to +0.61	0.112	0.746
RAAS inhibitor (ACE-I/ARB)	HR	216	73.41 ± 17.20	76.99 ± 19.40	−5.63 bpm	−11.02 to −0.24	0.041	0.406
RAAS inhibitor (ACE-I/ARB)	MAP	216	97.95 ± 14.19	96.48 ± 13.86	−0.77 mmHg	−4.66 to +3.12	0.696	0.906
RAAS inhibitor (ACE-I/ARB)	CI	216	2.90 ± 0.95	3.06 ± 1.00	−0.23 L/min/m²	−0.56 to +0.10	0.163	0.813
RAAS inhibitor (ACE-I/ARB)	SI	216	40.93 ± 13.21	41.35 ± 12.80	−0.23 mL/m²	−4.58 to +4.11	0.916	0.916
RAAS inhibitor (ACE-I/ARB)	TFC	138	32.80 ± 7.01	34.23 ± 8.67	−1.07 1/kΩ	−4.23 to +2.09	0.504	0.906
Calcium-channel blocker	HR	216	72.98 ± 18.98	76.04 ± 18.28	−3.10 bpm	−9.45 to +3.24	0.336	0.906
Calcium-channel blocker	MAP	216	97.57 ± 12.20	97.12 ± 14.37	−1.38 mmHg	−6.08 to +3.32	0.564	0.906
Calcium-channel blocker	CI	216	2.96 ± 1.25	2.99 ± 0.90	−0.10 L/min/m²	−0.49 to +0.28	0.600	0.906
Calcium-channel blocker	SI	216	42.44 ± 15.30	40.69 ± 12.26	+1.18 mL/m²	−4.03 to +6.40	0.655	0.906
Calcium-channel blocker	TFC	138	32.15 ± 7.14	34.01 ± 8.23	−1.92 1/kΩ	−5.38 to +1.54	0.274	0.906
Loop/thiazide(-like) diuretic	HR	225	75.66 ± 20.19	75.05 ± 17.27	−0.92 bpm	−6.61 to +4.77	0.751	0.906
Loop/thiazide(-like) diuretic	MAP	225	95.36 ± 13.82	97.93 ± 13.91	−4.36 mmHg	−8.38 to −0.35	0.033	0.406
Loop/thiazide(-like) diuretic	CI	225	2.98 ± 0.92	2.97 ± 0.98	−0.03 L/min/m²	−0.32 to +0.27	0.861	0.906
Loop/thiazide(-like) diuretic	SI	225	41.87 ± 14.81	40.59 ± 11.81	+1.78 mL/m²	−2.49 to +6.04	0.412	0.906
Loop/thiazide(-like) diuretic	SVRI	225	2968.81 ± 1676.04	2889.89 ± 1158.81	+39.95 dyn·s·cm^{-5}·m²	−385.10 to +465.00	0.853	0.906

Primary H1–H4 cells are reported in the main manuscript and were excluded from this 20-comparison FDR family. Raw group means are included for reporting transparency; inference is based on the prespecified adjusted HC3 models. All TFC models were restricted to CardioScreen/Niccomo observations; other outcomes included both acquisition systems.

Supplementary Table S4. Hemodynamic sensitivity analyses

Panel A. Primary H1–H4 sensitivity analyses

Hypothesis	Exposure → outcome	Analysis	N	Adjusted difference	95% CI	P	Classification
H1	Beta-blocker → HR1	Primary adjusted	214	+0.225	−5.126 to +5.576	0.934	Primary adjusted model
H1	Beta-blocker → HR1	+ admission NIHSS	214	+0.132	−5.261 to +5.525	0.962	Sensitivity analysis
H1	Beta-blocker → HR1	+ count of other primary medication classes	214	+0.768	−4.568 to +6.104	0.777	Sensitivity analysis
H1	Beta-blocker → HR1	+ NIHSS + count of other primary classes	214	+0.658	−4.739 to +6.055	0.810	Sensitivity analysis
H1	Beta-blocker → HR1	Exclude observations with Cook's D > 4/n	201	−1.822	−5.802 to +2.158	0.368	Influence sensitivity
H2	RAAS inhibitor → SVRI1	Primary adjusted	216	+191.731	−208.637 to +592.099	0.346	Primary adjusted model
H2	RAAS inhibitor → SVRI1	+ admission NIHSS	216	+192.206	−207.728 to +592.140	0.344	Sensitivity analysis
H2	RAAS inhibitor → SVRI1	+ count of other primary medication classes	216	+171.569	−235.967 to +579.104	0.407	Sensitivity analysis
H2	RAAS inhibitor → SVRI1	+ NIHSS + count of other primary classes	216	+179.064	−228.532 to +586.659	0.387	Sensitivity analysis
H2	RAAS inhibitor → SVRI1	Exclude observations with Cook's D > 4/n	210	+26.542	−256.744 to +309.827	0.854	Influence sensitivity
H3	CCB → SVRI1	Primary adjusted	216	+253.960	−278.595 to +786.515	0.348	Primary adjusted model
H3	CCB → SVRI1	+ admission NIHSS	216	+209.835	−310.955 to +730.624	0.428	Sensitivity analysis
H3	CCB → SVRI1	+ count of other primary medication classes	216	+227.083	−287.885 to +742.051	0.386	Sensitivity analysis
H3	CCB → SVRI1	+ NIHSS + count of other primary classes	216	+189.897	−319.406 to +699.200	0.463	Sensitivity analysis
H3	CCB → SVRI1	Exclude observations with Cook's D > 4/n	208	+181.973	−159.584 to +523.529	0.295	Influence sensitivity
H4	Loop/thiazide(-like) diuretic → TFC (CardioScreen/Niccomo only)	Primary adjusted	147	−3.717	−6.642 to −0.791	0.013	Primary adjusted model
H4	Loop/thiazide(-like) diuretic → TFC (CardioScreen/Niccomo only)	+ admission NIHSS	147	−3.219	−6.170 to −0.268	0.033	Sensitivity analysis
H4	Loop/thiazide(-like) diuretic → TFC (CardioScreen/Niccomo only)	+ count of other primary medication classes	147	−3.255	−6.288 to −0.221	0.036	Sensitivity analysis
H4	Loop/thiazide(-like) diuretic → TFC (CardioScreen/Niccomo only)	+ NIHSS + count of other primary classes	147	−2.634	−5.772 to +0.505	0.099	Sensitivity analysis
H4	Loop/thiazide(-like) diuretic → TFC (CardioScreen/Niccomo only)	Exclude observations with Cook's D > 4/n	139	−2.961	−5.400 to −0.522	0.018	Influence sensitivity

Panel B. Secondary models with additional admission NIHSS adjustment

Exposure	Outcome	N	Adjusted difference	95% CI	P	BH-FDR q
Systemic beta-blocker	MAP	214	−1.52 mmHg	−5.45 to +2.40	0.445	0.970
Systemic beta-blocker	CI	214	+0.04 L/min/m^2	−0.30 to +0.38	0.821	0.970
Systemic beta-blocker	SI	214	+0.58 mL/m^2	−3.64 to +4.81	0.785	0.970
Systemic beta-blocker	SVRI	214	+118.87 dyn·s·cm^{-5}·m^2	−333.22 to +570.95	0.605	0.970
Systemic beta-blocker	TFC	137	−2.84 1/kΩ	−6.04 to +0.37	0.083	0.551
RAAS inhibitor (ACE-I/ARB)	HR	216	−5.64 bpm	−11.00 to −0.27	0.039	0.398
RAAS inhibitor (ACE-I/ARB)	MAP	216	−0.78 mmHg	−4.67 to +3.12	0.695	0.970
RAAS inhibitor (ACE-I/ARB)	CI	216	−0.23 L/min/m^2	−0.57 to +0.10	0.164	0.818
RAAS inhibitor (ACE-I/ARB)	SI	216	−0.23 mL/m^2	−4.59 to +4.13	0.916	0.970
RAAS inhibitor (ACE-I/ARB)	TFC	138	−0.81 1/kΩ	−3.86 to +2.24	0.600	0.970
Calcium-channel blocker	HR	216	−2.88 bpm	−9.35 to +3.59	0.381	0.970
Calcium-channel blocker	MAP	216	−1.28 mmHg	−6.02 to +3.46	0.596	0.970
Calcium-channel blocker	CI	216	−0.08 L/min/m^2	−0.46 to +0.30	0.680	0.970
Calcium-channel blocker	SI	216	+1.36 mL/m^2	−3.86 to +6.58	0.609	0.970
Calcium-channel blocker	TFC	138	−1.61 1/kΩ	−5.15 to +1.92	0.368	0.970
Loop/thiazide(-like) diuretic	HR	225	−0.54 bpm	−6.32 to +5.24	0.853	0.970
Loop/thiazide(-like) diuretic	MAP	225	−4.29 mmHg	−8.37 to −0.20	0.040	0.398
Loop/thiazide(-like) diuretic	CI	225	−0.01 L/min/m^2	−0.31 to +0.29	0.951	0.970
Loop/thiazide(-like) diuretic	SI	225	+1.82 mL/m^2	−2.52 to +6.17	0.409	0.970
Loop/thiazide(-like) diuretic	SVRI	225	−7.72 dyn·s·cm^{-5}·m^2	−418.21 to +402.77	0.970	0.970

Panel C. Influence sensitivity for nominal secondary signals

Exposure	Outcome	Base N	Base β / P / q	Excluded	Refit N	Refit β (95% CI)	Refit P
RAAS inhibitor (ACE-I/ARB)	HR	216	−5.63 bpm; P=0.041; q=0.406	15	201	−4.91 bpm (−8.75 to −1.08)	0.012
Loop/thiazide(-like) diuretic	MAP	225	−4.36 mmHg; P=0.033; q=0.406	13	212	−2.10 mmHg (−5.59 to +1.39)	0.237

For each H1–H4 model, the other-medication-class count was defined as the sum of binary exposure indicators for the remaining three of the four primary medication classes (systemic beta-blockers, RAAS inhibitors, calcium-channel blockers, and loop/thiazide(-like) diuretics), excluding the index exposure. This explicitly defined count was used in the revised sensitivity analyses. Sensitivity analyses do not replace the primary or FDR-controlled secondary inference. All H4 and other TFC models were restricted to CardioScreen/Niccomo observations because technical equivalence of absolute TFC output across the two proprietary platforms was not established.

Supplementary Table S5. Six-month mortality: raw contrasts and sensitivity analyses

Panel A. Raw mortality contrasts and adjusted models

Exposure	Raw exposed	Raw unexposed	Model 1 OR (95% CI)	P/q	Principal Model 2 OR (95% CI)	P/q	N/deaths
Beta-blocker	27/88 (30.7%)	20/120 (16.7%)	1.44 (0.69–3.00)	0.333/0.766	1.22 (0.52–2.84)	0.646/0.806	197/47
RAAS inhibitor	21/98 (21.4%)	26/110 (23.6%)	0.86 (0.40–1.83)	0.695/0.766	0.76 (0.31–1.86)	0.546/0.806	202/47
CCB	7/47 (14.9%)	40/161 (24.8%)	0.36 (0.14–0.95)	0.038/0.231	0.41 (0.13–1.26)	0.119/0.711	196/47
Loop/thiazide diuretic	14/63 (22.2%)	33/145 (22.8%)	0.74 (0.33–1.66)	0.467/0.766	0.82 (0.32–2.10)	0.672/0.806	202/47
Statin	18/78 (23.1%)	29/130 (22.3%)	0.84 (0.39–1.80)	0.660/0.766	0.80 (0.32–2.00)	0.636/0.806	194/47
ASA	20/86 (23.3%)	27/122 (22.1%)	0.89 (0.41–1.94)	0.766/0.766	1.02 (0.40–2.58)	0.968/0.968	189/44

Panel B. Inverse-probability-of-observation weighted sensitivity

Weights were estimated separately for each medication exposure in the full medication-analysis cohort (n=233). The denominator model used L2-penalized logistic regression (ridge, C=1; age and NIHSS standardized for fitting) for observed 6-month follow-up, including exposure, age, sex, admission NIHSS, and three-center information, to obtain finite estimates despite complete follow-up in one center; the numerator model included exposure alone. The same weighted-outcome models were repeated over C=0.1, 1, 10, and 100 to assess sensitivity to penalization strength. No weight truncation was applied. Mean and maximum weights shown below are calculated among participants in the weighted outcome model with observed follow-up. Weighted outcome models retained the principal mortality covariates and used sandwich (HC0) robust standard errors treating the estimated weights as fixed; these intervals do not incorporate uncertainty from estimating the observation model. No exposure association met $P<0.05$ across the tested penalty strengths. As an additional uncertainty assessment, a center-stratified patient-level bootstrap was performed (400 replicates per exposure); in each replicate, the ridge-penalized observation model, unpenalized exposure-only numerator, resulting weights, and weighted mortality model were refitted. All 400 replicates were estimable for each exposure. Percentile bootstrap 95% CIs for the weighted ORs were: beta-blockers 0.51–3.13; RAAS inhibitors

0.28–1.70; CCBs 0.06–1.29; loop/thiazide(-like) diuretics 0.29–2.08; statins 0.24–2.18; ASA 0.34–3.20. All intervals included 1. The table reports the original model-based HC0 confidence intervals and P values; the supplementary bootstrap intervals additionally incorporate the re-estimation of observation weights. The bootstrap does not remove possible selection bias or confounding.

Exposure	N/deaths	Mean weight	Max weight	Weighted OR	95% CI	P
Beta-blocker	197/47	1.003	1.221	1.28	0.56–2.90	0.557
RAAS inhibitor	202/47	1.001	1.238	0.74	0.32–1.74	0.494
CCB	196/47	1.000	1.230	0.43	0.13–1.44	0.172
Loop/thiazide diuretic	202/47	1.001	1.227	0.83	0.33–2.12	0.700
Statin	194/47	1.002	1.233	0.78	0.31–2.00	0.607
ASA	189/44	1.001	1.282	1.07	0.41–2.80	0.883

Panel C. Extreme-case assignment of unknown outcomes

Exposure	Scenario	N	Deaths	OR	95% CI	P
Beta-blocker	All unknown = alive	221	47	1.16	0.51–2.63	0.722
Beta-blocker	All unknown = dead	221	71	1.10	0.55–2.19	0.794
RAAS inhibitor	All unknown = alive	226	47	0.67	0.29–1.58	0.364
RAAS inhibitor	All unknown = dead	226	71	0.83	0.41–1.66	0.591
CCB	All unknown = alive	221	47	0.48	0.16–1.40	0.176
CCB	All unknown = dead	221	72	0.36	0.15–0.88	0.026
Loop/thiazide diuretic	All unknown = alive	226	47	0.95	0.38–2.32	0.903

Exposure	Scenario	N	Deaths	OR	95% CI	P
Loop/thiazide diuretic	All unknown = dead	226	71	1.03	0.49–2.18	0.928
Statin	All unknown = alive	219	47	0.88	0.38–2.08	0.775
Statin	All unknown = dead	219	72	0.86	0.42–1.76	0.670
ASA	All unknown = alive	214	44	1.10	0.46–2.63	0.827
ASA	All unknown = dead	214	69	1.00	0.49–2.06	0.999

Panel D. Non-linear age sensitivity

Exposure	N	Age non-linearity P	NIHSS non-linearity P	Age-spline OR	95% CI	Drug P
Beta-blocker	197	0.010	0.546	1.15	0.48–2.79	0.751
RAAS inhibitor	202	0.012	0.663	0.82	0.32–2.09	0.675
CCB	196	0.002	0.563	0.41	0.13–1.31	0.134
Loop/thiazide diuretic	202	0.011	0.642	0.80	0.30–2.15	0.663
Statin	194	0.002	0.327	0.85	0.33–2.22	0.743
ASA	189	0.004	0.583	0.84	0.31–2.26	0.733

Model 1 uses prespecified indication-related covariates. Principal Model 2 additionally adjusts for admission NIHSS. BH-FDR correction applies across the six medication exposures within each principal model family.

Supplementary Table S6. Exploratory pre-stroke anticoagulation analyses among patients with atrial fibrillation

Exposure definition	Model	N/deaths	Exposed mortality	Unexposed mortality	Adjusted OR (95% CI)	P
Dedicated AF anticoagulation field	Age + sex	67/26	10/34 (29.4%)	16/33 (48.5%)	0.55 (0.19–1.59)	0.272
Dedicated AF anticoagulation field	Age + sex + NIHSS	67/26	10/34 (29.4%)	16/33 (48.5%)	0.64 (0.19–2.14)	0.473
Medication-list canonical anticoagulant	Age + sex	67/26	10/32 (31.2%)	16/35 (45.7%)	0.67 (0.23–1.92)	0.455
Medication-list canonical anticoagulant	Age + sex + NIHSS	67/26	10/32 (31.2%)	16/35 (45.7%)	0.76 (0.23–2.51)	0.651

The dedicated AF anticoagulation field was the prespecified primary exposure definition. Medication-list canonical anticoagulant coding was used as a sensitivity definition. These analyses are exploratory.
